# Effect of Chemical Modification on Mechanical Properties of Wood-Plastic Composite Injection-Molded Parts

**DOI:** 10.3390/polym10121391

**Published:** 2018-12-15

**Authors:** Joo Seong Sohn, Sung Woon Cha

**Affiliations:** School of Mechanical Engineering, Yonsei University, 50, Yonsei-ro, Seodaemun-gu, Seoul 03722, Korea; ssamjjang87@yonsei.ac.kr

**Keywords:** wood-plastic composite, chemical modification, co-rotating intermeshing twin-screw extrusion, composite pellet, injection molding

## Abstract

Wood chips from furniture-manufacturing byproducts, which do not include adhesive or paint in the waste wood, were used for the flouring process and chemical modification of wood flour (WF). After chemical modification, the WF was mixed with polypropylene through extrusion compounding and injection-molding to prepare wood-plastic composite (WPC) injection-molded specimens for the American Society for Testing and Materials. Static contact angle measurements and stereomicroscope observations were performed. In this study, it was confirmed that the impact strength was improved by up to 55.8% and the tensile strength by up to 33.8%. The flexural modulus decreased marginally. As a result of WF chemical modification, the measured contact angle of WPC increased, which means that the wettability of the WPC specimen surface decreased. In addition, it was observed through stereomicroscopy that the whitening of the surface of the WPC specimen improved.

## 1. Introduction

Wood-plastic composite (WPC) is a composite material made of wood fiber/wood flour (WF) and thermoplastic(s). It is primarily used for decking, railings, cladding, sidings, and window and door frames, and can be molded to approximately any desired shape. It exhibits superior characteristics compared to existing processed wood products; for example, it is highly resistant to rot, decay, and corrosion. In addition, it is advantageous in terms of its specific gravity and manufacturing costs compared to glass fiber reinforced composite materials and is commonly used as a plastic reinforcement material. Because of the ability of WPC to be molded to meet almost any desired shape, both the use ratio and market share of WPC are gradually increasing. In addition, efforts are being made to reduce WPC processing costs through the use of recycled plastics and waste wood (as opposed to expensive imported wood chips). 

Recently, studies have been conducted to improve the quality and properties of injection-molded WPC products [1,2]. In these studies, attempts were made to improve the interfacial adhesion between the WF and polymer resin. Hydrophilic wood and hydrophobic polymer resins have different properties, which decrease interfacial adhesion and reduce mechanical properties. Therefore, numerous studies on the chemical modification of WF using various pretreatment methods and coupling agents have been reported.

These studies typically improved the compatibility of hydrophilic wood, as it permanently reforms its molecular structure by chemical modification of WF [3,4,5,6].

This study investigated the effect of chemical modification of WF on the mechanical properties of WPC injection-molded parts. It considered to be a WPC made of WF and polypropylene (PP). The WF was fabricated from paint- and adhesive-free wood chips that were collected during a particle board and medium-density fiber board manufacturing process (that is, the WF was fabricated from waste wood). The fabricated WF first underwent chemical modification before being mixed with PP, by way of an extrusion compounding process, to make the desired WPC. The process of chemical modification served to improve the interfacial adhesion between the PP matrix and the WF of the WPC and made use of a compatibilizer, maleic anhydride-grafted polypropylene (MAPP), and silane as a coupling agent. The WPC strands formed through the extrusion compounding process were transported along a conveyor belt to a cutting machine, where they were subsequently cut into pellets. The use of a conveyor belt as a means of cooling the WPC strands was preferred to that of water cooling because the hydroxyl groups present on the surface of wood flour are hydrogen bonded, which could cause defective molding as the water absorption rate increases. The pellets were then re-melted and formed into WPC injection-molded American Society for Testing and Materials (ASTM) specimens, of which the mechanical properties and formability were investigated. In particular, the moldability of the specimens was evaluated by measuring the impact strength (ASTM D-256), tensile strength (ASTM D-638), and flexural modulus (ASTM D-790). The mechanical properties and surface properties of the WPC specimens made from chemically modified WF and WPC specimens made from untreated WF were compared by evaluating their moldability. The tensile strength and flexural modulus were measured using a universal testing machine (QMESYS Co., Ltd., Gunpo, Korea, Model No. QM-100T), and impact strength was measured using a digital impact tester (Salt Co., Ltd., Incheon, Korea, Model No. ST-120). Seven specimens were produced for the set injection molding process conditions, and five specimens were used to calculate the average value for each mechanical property. The highest and lowest values in a set of seven specimens were discarded and the mean value was calculated.

## 2. Materials and Methods

**Figure 1:**
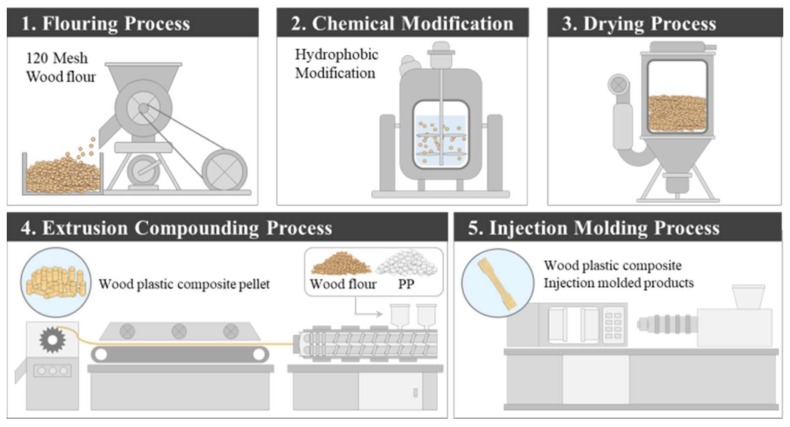
A schematic of the experimental process.

Summarizing the experimental process in Figure 1, the wood chips (*Pinus bungeana*, density = 100–135 g/L) are classified into 120 mesh (ISO particle size = 125 μm, 120 openings per 2.54 cm^2^) through the flouring process. Hemicellulose, lignin, and tannin, which do not contribute to the mechanical properties, were then removed by alkali pretreatment. In particular, lignin is degraded by ultraviolet rays, which could cause surface quality deterioration because of the whitening phenomenon. The modified WF is dried and then put into a twin-screw extruder and molded into pellets. Finally, the pellets are re-melted by an injection molding machine to produce a specimen.

### 2.1. Equipment

In this study, a co-rotating intermeshing twin-screw extruder (BAUTEK Inc., Pocheon, Korea, Product no. BA-19) with a screw *L*/*D* of 40 was used to prepare composite pellets. An injection-molding machine (LG Electronics Inc., Changwon, Korea, LGH-100N) with a screw *L*/*D* of 28 was used to prepare the WPC injection-molded ASTM specimens.

The WPC strand produced by the twin-screw extruder was air cooled using a conveyor belt. General plastic resin is conventionally water cooled. However, WF is prone to absorb moisture by hydrogen bonding by hydrophilic hydroxyl functional groups on its surface. This could result in voids in the molded product because of the residual moisture of the pellets when the molded product is later produced, which could cause deterioration of the mechanical performance.

### 2.2. Materials

#### 2.2.1 Base Resin and Additive

Polypropylene (POLYPIA Inc., Dangjin, Korea, product grade no. BE1451S, melt index = 16 g/10 min) and wood chips (supplied by SINWOO TNC Inc. Incheon, Korea, and collected at 120 mesh during a particle board and medium-density fiber board manufacturing process) were used in the fabrication of the WPC.

Maleic anhydride-grafted polypropylene (at 3 parts per hundred resin (phr)) (EASTMAN Inc., Kingsport, TN, USA, product grade no. G-3003) was added to the extrusion compounding process to improve the compatibility of the WF and the PP used in the fabrication of the WPC.

#### 2.2.2 Chemical Reagents Methods

Alkali reagent (NaOH, MW = 39.997 g/mol, density = 2.13 g/cm^3^, boiling point = 1,388 ℃) was used as a pretreatment agent for removing impurities, and ethanol (C_2_H_5_OH, MW = 46.07 g/mol, density = 0.79 g/cm^3^, boiling point = 78.37 ℃) was used for hydrolysis of the silane reagent. Both reagents were purchased from Duksan Pure Chemicals Co., Ltd., Ansan, Korea, Vinyltrimethoxysilane (VTMS) (H_2_C = CHSi(OCH_3_)_3_, MW = 148.23 g/mol, boiling point = 125 ℃) was used as a silane coupling agent and was purchased from Tokyo Chemical Industry Co., Ltd., Tokyo, Japan.

#### 2.2.3. Chemical Modification of Wood Flour

**Figure 2:**
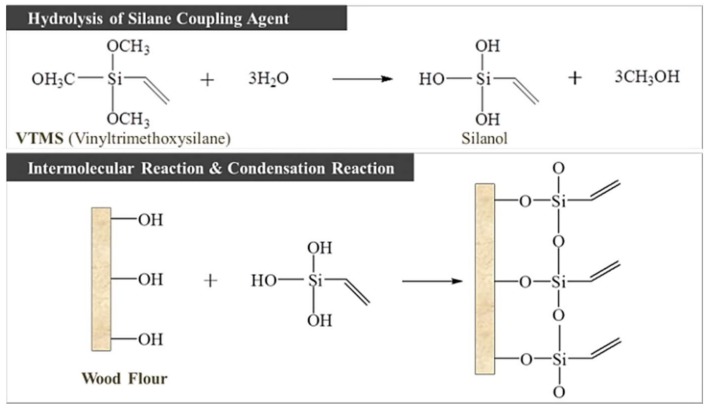
The mechanism for hydrophobic modification reaction of wood flour (WF).

In the chemical modification process (Figure 2), WF was added to the chemical treatment agitator, and the primary agitation process was performed at room temperature for 1 h in a 2.5% NaOH aqueous solution. The ratio of the NaOH aqueous solution and the WF ratio for the alkali pretreatment was 20:1 (wt %) [7,8,9,10]. The alkali pretreatment with NaOH serves to remove any impurities present on the surfaces of the wood chips (waste wood) as well as any elements that do not contribute to the physical properties of the wood chips, such as lignin, tannin, and ash [11]. In addition, WF exhibiting hydrophilic properties by the OH functional group on the cellulose surface is required to be modified to improve compatibility with hydrophobic olefinic plastic raw materials. For this purpose, 2.5% VTMS was added to a 60:40 mixture of ethanol and water to perform silane coupling for the hydrophobic modification of hydrophilic WF, followed by a secondary agitation process at room temperature for 2 h. After the chemical modification, the WF was dried at a temperature of 90 °C for 12 h.

#### 2.2.4. The Processing Method of Wood-Plastic Composite

**Figure 3:**
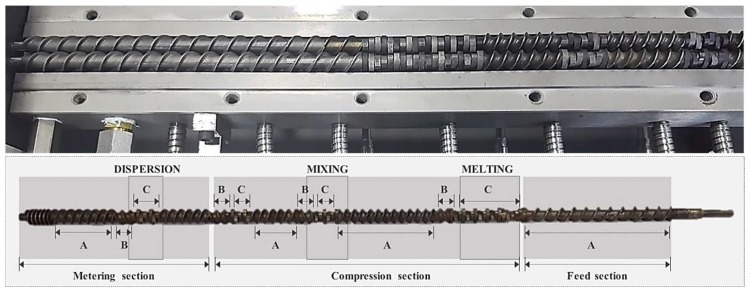
The configuration of co-rotating intermeshing twin-screw (A = Forward conveying screw elements, B = Reverse conveying screw elements, C = Kneading screw elements).

**Figure 4:**
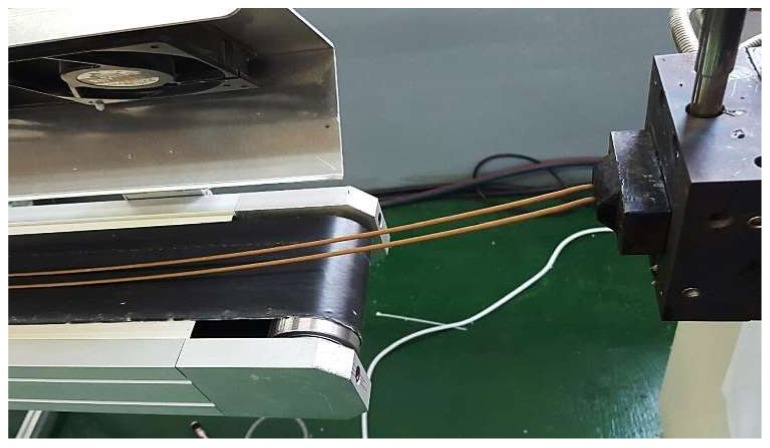
Extrusion compounding process of the wood-plastic composite.

Wood flour, PP, and MAPP were homogeneously mixed by a mixer and fed into the hopper of a co-rotating intermeshing twin-screw (Figure 3) extruder as part of the extrusion compounding process (Figure 4). At this time, PP and WF were mixed at weight mixing ratios of 90:10, 80:20, 70:30, 60:40, and 50:50, and MAPP was added in the amount of 3 phr based on the weight of the mixture of WF and PP (Table 1).

**Figure 5:**
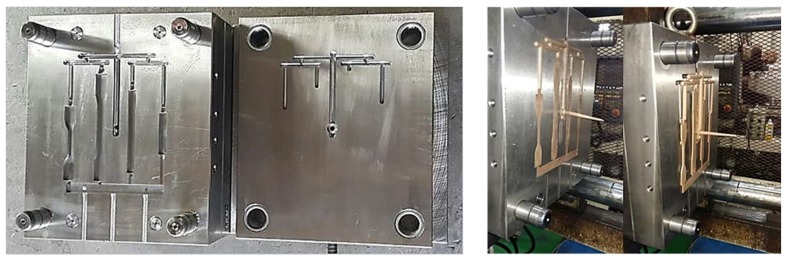
The injection molding American Society for Testing and Materials (ASTM) standard test mold used in this study.

**Figure 6:**
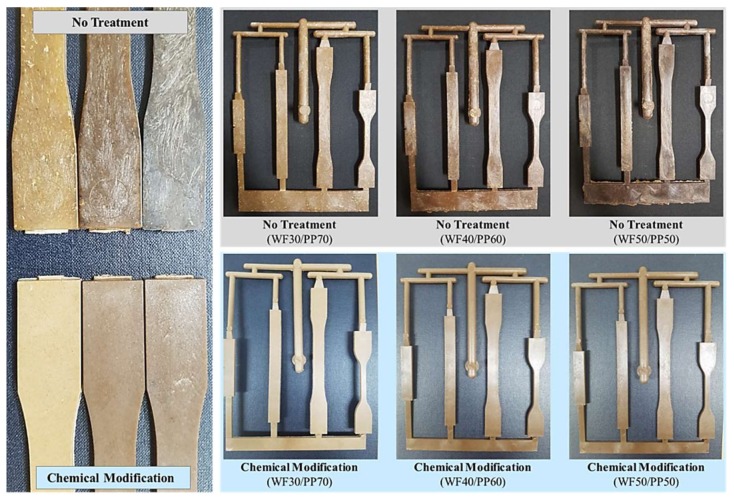
Chemically modified and untreated wood-plastic composite injection-molded ASTM specimens.

Finally, injection-molded ASTM specimens for the measurement of mechanical properties were prepared using compounded composite pellets (Figure 5 and Figure 6). The experimental conditions of the extrusion compounding process are presented in Table 2, and those of the injection molding process are presented in Table 3. The injection temperature did not exceed 200 °C in order to prevent the loss of mechanical strength because of thermal deformation of the cellulose molecules in the WF. The compounding temperature of the extruder was set at 160 °C. This is the lowest temperature at which the base material PP can be melted to prepare a composite material pellet. However, the nozzle temperature of the injection molding machine was set at 190 °C, which is marginally greater than the temperature of the extruder, in order to ensure sufficient fluidity of the composite material melt in the mold and to prevent carbonization of the natural material.

## 3. Results and Discussion

### 3.1 The Changes in Surface Chemical Composition of Wood Flour

**Figure 7:**
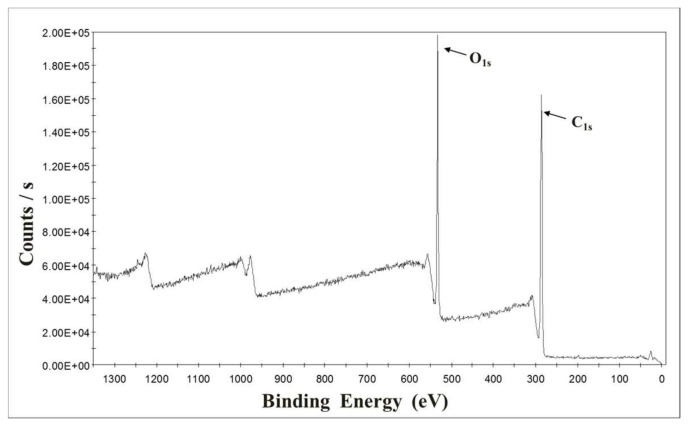
XPS spectra of untreated wood flour.

**Figure 8:**
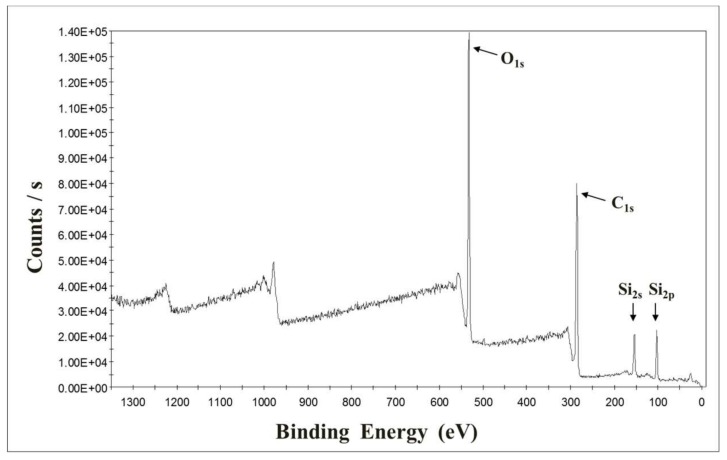
XPS spectra of chemically modified wood flour.

X-ray photoelectron spectroscopy (XPS, model no. K-alpha, Thermo Scientific Inc., Leicestershire, UK) measurements were performed to investigate the hydrophobic modification effect of silanol grafting on the surface of wood flour. XPS spectra used Al Kα (*hν*=1486.6 eV) radiation to record the surface of untreated wood flour and chemically modified wood flour samples. Figure 7 and Figure 8 present the XPS spectra of untreated wood flour and chemically modified wood flour, respectively. The figures show the number of electrons counted versus the binding energy in electron volts (eV). The untreated wood flour showed no Si_2s_, Si_2p_ peaks and only C_1s_ and O_1S_ peaks were observed. The Si_2s_, Si_2p_ peaks were observed in the chemically modified wood flour, indicating that the silane functional group was included on the surface (mainly peaks related to SiC, SiO_2_ and SiO_2_C_2_ bonds are reported on the surface.) The elemental compositions of all the studied spectra are summarized in Table 4 as the relative percentages of the elements. It is seen that the contents of Si are increased remarkably after chemical modification. Therefore, it can be confirmed that the VTMS is hydrolyzed and grafted onto the wood flour surface to change the chemical composition.

### 3.2. Impact Strengths of Chemically Modified and Untreated Wood-Plastic Composite Injection-Molded ASTM Specimens

**Figure 9:**
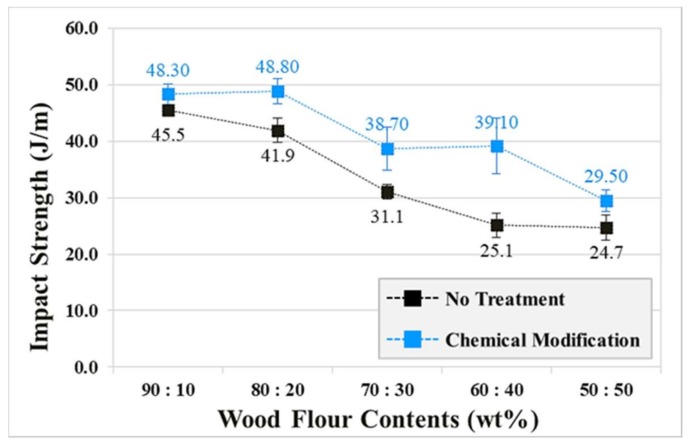
Impact strengths of chemically modified and untreated wood-plastic composite injection-molded ASTM specimens.

Impact strength was measured in accordance with the ASTM D 256 method, and the specimen sizes were 63.5 × 12.7 × 3.0 (mm^3^). The impact was applied to the side of the notch [12]. Impact strengths were calculated after five repeated measurements. The results (Figure 9) exhibited a tendency for the impact strengths to decrease in both the chemically modified and untreated cases as the mixing ratio approached 50:50. The impact strengths of the chemically modified specimens improved by at least 6.2%, which was 55.8% greater than that of the untreated specimens. The chemically modified specimens exhibited higher levels of impact strength than the untreated specimens across all considered mixing ratios. The reason for the high level of impact strength in the chemically modified specimens is the increased interfacial adhesion between the WF and the PP. In the case of the untreated specimens, the interfacial adhesion between the WF and PP would be low, resulting in aggregation of the WF and reduced dispersibility. This creates a crack initiation site [13].

### 3.3. Tensile Strength of Chemically Modified and Untreated Wood-Plastic Composite Injection-Molded ASTM Specimens

**Figure 10:**
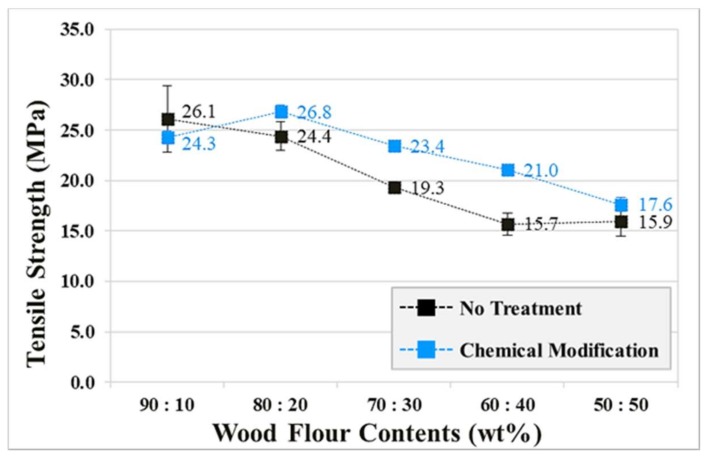
Tensile strengths of chemically modified and untreated wood-plastic composite injection-molded ASTM specimens.

The tensile strength was measured in accordance with the ASTM D 638 method, and the specimen sizes were 165 × 19 × 3.3 (mm^3^). The cross-head speed was 10 mm/min when measuring the tensile strength [14]. Tensile strengths were calculated after five repeated measurements. The tensile strengths of the specimens (Figure 10) exhibited the tendency to decrease in both the chemically modified and untreated cases as the mixing ratio approached 50:50. The tensile strengths of the chemically modified specimens improved by at least 10.7%, which was 33.8% greater than that of the untreated specimens. The chemically modified specimens exhibited higher levels of tensile strength than the untreated specimens across all but one of the considered mixing ratios (PP90:WF10). In the case of the untreated specimens, wood fibers of the WF did not function correctly as reinforcement. This is because elements that did not contribute to the physical properties had not been removed. Alkali pretreatment removes elements such as hemicellulose, lignin, extractives, and wax, and also makes the surface of WF softer and fibrous. Elimination of these factors improves the mechanical properties of the WPC [15,16,17,18,19,20,21,22]. Conversely, we found that the chemical modification of WF improved the tensile strength of the chemically modified specimens as the interfacial interactions between the PP matrix and the hydrophobized WF were enhanced by removing impurities that would interfere with the compatibility between the dissimilar materials [23].

In addition, complete adhesion of the chemically modified WF and PP matrix was confirmed from the scanning electron microscope images from previous studies in this regard [24].

### 3.4. Flexural Modulus of Chemically Modified and Untreated Wood-Plastic Composite Injection-Molded ASTM Specimens

**Figure 11:**
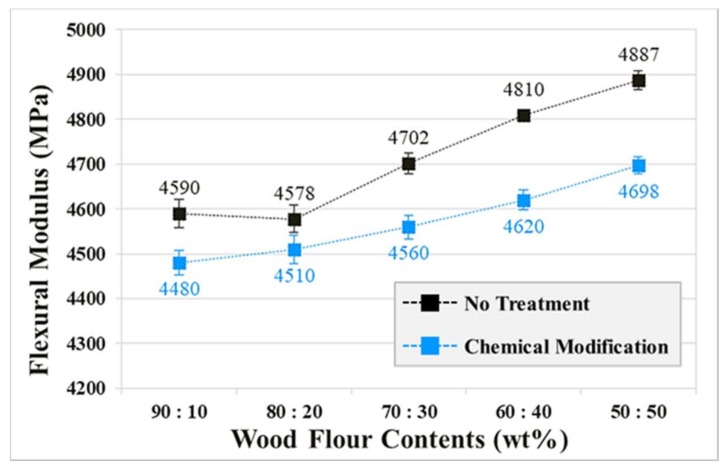
The flexural modulus of chemically modified and untreated wood-plastic composite injection-molded ASTM specimens.

The flexural modulus was measured in accordance with the ASTM D 790 method, and the specimen sizes were 127 × 12.7 × 6.4 (mm^3^). The support distance of specimens was 102.4 mm and the test load speed was 10 mm/min [25]. Flexural moduli were calculated after five repeated measurements. The results of the WPC specimens (Figure 11) exhibited a tendency to increase in both the chemically modified and untreated cases as the mixing ratio approached 50:50. The untreated specimens exhibited a greater flexural modulus than the chemically modified specimens across all of the considered mixing ratios. The two experimental conditions (untreated and treated WF) exhibited increasing flexural moduli with decreasing ductility as the WF content increased. The results of this experiment also indicate that chemical modification of WF cannot be expected to improve the flexural modulus. 

### 3.5. Contact Angle of Chemically Modified and Untreated Wood-Plastic Composite Injection-Molded ASTM Specimens

**Figure 12:**
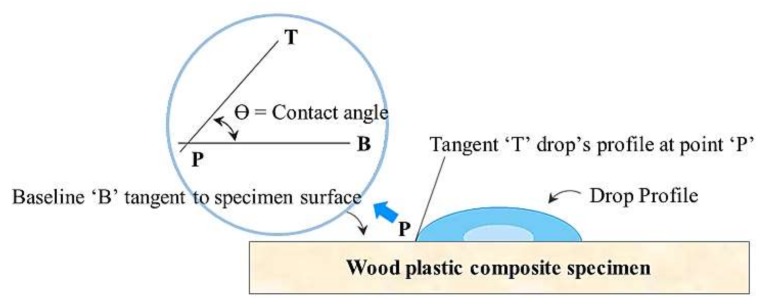
The contact angle measurement method used in this study.

**Figure 13:**
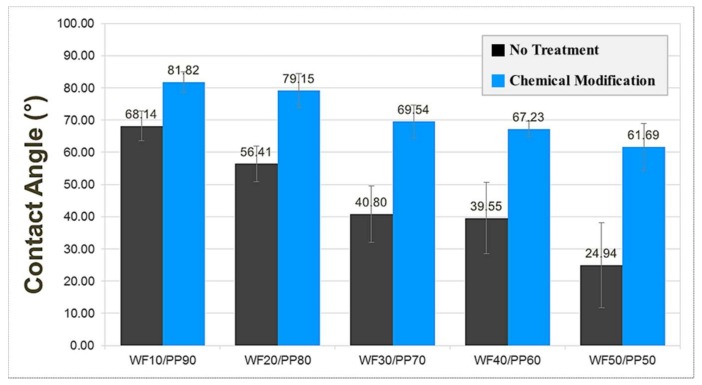
The contact angle measurements of chemically modified and untreated wood-plastic composite injection-molded ASTM specimens.

**Figure 14:**
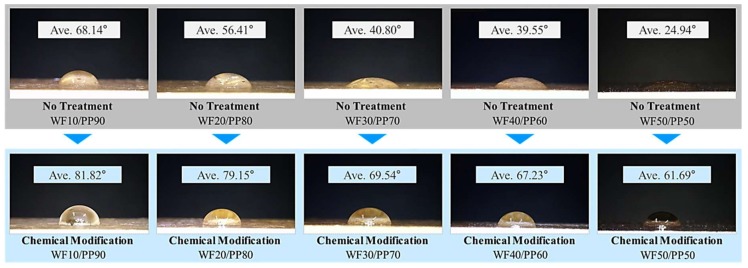
Contact angle measurements of chemically modified and untreated wood-plastic composite injection-molded ASTM specimens.

The contact angle, which is the angle between the sessile droplet and the surface of the specimen, was measured by a micro-pipette (model no. AP-10, AXYGEN Inc., Union City, CA, USA) at room temperature and humidity of 50% after dropping 3 μL of distilled water on the surface of the tensile strength specimen using a digital microscope (Dino-Lite, model no. AM4113ZT, AnMo electronics Corp., New Taipei City, Taiwan) (Figure 12). Contact angle measurements on the surfaces of WPC injection-molded ASTM specimens were performed to investigate the effect of chemical modification on hydrophilic WF. The measured contact angles of the chemically modified specimens (Figure 13 and Figure 14) were significantly greater than those of the untreated specimens. The contact angle decreased with increasing WF content in the cases both with and without chemical modification. Considering that the surface contact angle of pure PP was 97°, the surface characteristics of the ASTM specimen with a WF content of 50 wt % exhibited hydrophilic surface characteristics with a significantly high wettability. As the amount of WF increases, further studies on the silane concentration will be required.

### 3.6. Evaluation of Surface Quality of WPC Specimen by Chemical Modification of Wood Flour

Stereomicroscope (Nikon Inc., Tokyo, Japan, product no. SMZ 1270) observations confirmed the reduction of the whitening phenomenon by lignin. We believe this is because of the dissolution of lignin by alkali pretreatment. The hydrophobic modification by silane also contributed to the improvement of dispersibility by improving the compatibility of WF with PP.

## 4. Conclusions

The aim of this study was to improve the interfacial adhesion between the PP matrix and the WF of WPC by inducing hydrophobic modification of WF by applying an alkali treatment and silane coupling process for the chemical modification of WF. The chemical modification process is a method for generating a new chemical bond between silane and the PP matrix as a means of improving interfacial adhesion between the PP matrix and the WF of the WPC. The results of the chemical modification process according to the WF content in this study are summarized as follows.

The WPC injection-molded ASTM specimens with improved interfacial adhesion exhibited a maximum increase of 55.8% of the impact strength and 33.8 % of the tensile strength. The flexural modulus of the untreated WPC injection-molded ASTM specimens was greater than that of the chemically modified WPC injection-molded ASTM specimens across all of the considered mixing ratios. However, the flexural modulus of the chemically modified WPC injection-molded ASTM specimens exhibited a tendency to increase as the mixing ratio approached 50:50. This was because the alkali treatment process negatively affected the elastic modulus of the WF.

**Figure 15:**
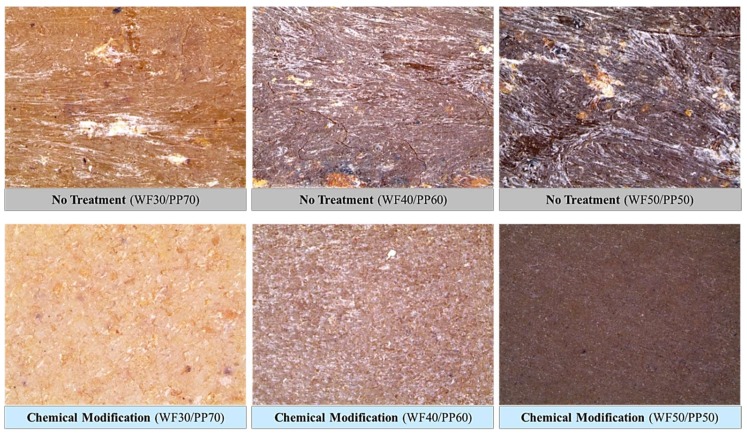
Stereomicroscope observation (magnification: 80 ×) of chemically modified and untreated wood-plastic composite injection-molded ASTM specimens.

As a result of chemical modification of the WF, the wettability decreased marginally as the contact angle of the surface of the WPC composite specimen increased. In addition, from stereomicroscopic observations, the lignin was removed by the alkali pretreatment during the chemical modification process, and the surface whitening phenomenon decreased (Figure 15).

## Figures and Tables

**Table 1:** Material preparation conditions.

Material	Polypropylene	Wood Flour
Chemical modification	-	NaOH 2.5%/VTMS 2.5%
Mixing ratio (wt %)	PP:WF = 90:10/80:20/70:30/60:40/50:50	
Drying time (h)	-	12 (90 ℃)
Particle size (mesh)	-	120
Compatibilizer (phr)	Maleated polypropylene (3)	

**Table 2:** Co-rotating intermeshing twin-screw extruder settings.

Co-Rotating Twin-Screw Extruder Machine (BA-19)								
Extrusion temp. (℃)	HD	H1	H2	H3	H4	H5	H6	H7
	160	160	150	150	150	150	140	130
Die diameter (Ф)	3							
Press. (MPa)	5.5							
Screw rpm	120							
Throughput (kg/h)	5							

**Table 3:** Injection molding machine settings.

Injection Molding Machine (LGH-100N)					
Injection temp. (℃)	HD	H1	H2	H3	H4
	190	190	180	170	160
Injection press. (MPa)	5				
Injection speed (mm·s^−1^)	80				
Back press. (MPa)	1				
Holding press. (MPa)	4				
Holding time (s)	3				
Mold temp. (℃)	40				
Cooling time (s)	30				
Screw rpm	100				

**Table 4:** The surface elemental composition ratio of untreated wood flour and chemically modified wood flour by XPS measurement.

Elements	No Treatment		Chemical Modification	
	Percent (%)	Ratio to C (%)	Percent (%)	Ratio to C (%)
C	74.67	100.00	58.45	100.00
O	25.06	33.56	29.75	50.98
Si	0.27	0.36	11.80	20.19
